# Beyond travel restrictions: exploring alternative infection control measures for Japanese medical students

**DOI:** 10.5116/ijme.64b8.db07

**Published:** 2023-07-31

**Authors:** Yudai Kaneda, Miyuka Kushibe

**Affiliations:** 1 School of Medicine, Hokkaido University, Hokkaido, Japan; 2 School of Medicine, University of Debrecen, Debrecen, Hungary

**Keywords:** COVID-19, Travel Restriction, Medical Students, Japan

## To the Editor

In the current context, where the Omicron variant has become the dominant strain of the novel coronavirus infection (COVID-19), the effectiveness of travel restrictions is increasingly being questioned.^1^ In fact, in Japan, since October 11, 2022, the "Guidelines for Accepting Foreign Tourists" have been abolished, and PCR testing upon entry is no longer mandatory. On the other hand, the reality is that medical students still face restrictions on their movement. In many Japanese medical schools, students participating in hospital internships must report to the medical faculty and obtain permission if they plan to travel outside the prefecture, even if the purpose is to prepare for employment through hospital observation tours.^2^ They are required to provide information regarding the mode of transportation used, the purpose of the visit, and the city where they will stay, all of which should be reported within a specified deadline of one to two weeks before their departure.^2^ As a result, some students have faced disciplinary action for moving without proper application.

Although discussions on individual rights, including movement restrictions and freedom of career choice, are often overlooked in Japan's medical schools, it is essential to note that these are personal rights recognized by the Japanese constitution.^3^^, ^^4^ While the norms established by the constitution are primarily designed to regulate the state, as Japan is a constitutional state, these norms must be respected in all contexts, including contracts between medical schools and students. Ideally, when restricting individual rights, it is necessary to evaluate the appropriateness of the restriction based on the concept of public welfare, which requires a discussion of the plausibility of the purpose and means.^5^

The purpose of the current measure is to prevent students participating in hospital internships from spreading COVID-19 to hospital staff, including patients. Given the frequent occurrence of clusters in hospitals and considering the risks to patients,^6^ this objective can be deemed highly reasonable. However, the issue lies in the means employed. The measure aims to control the spread of infection by "managing travel outside the prefecture." However, since COVID-19 is known to cause aerosol transmission, and since there have been cases of infection in every prefecture in Japan, there is little scientific rationale or evidence to support this to the best of our knowledge regarding the effectiveness of movement control as an infection control measure for individuals.^7^ This is because infections occur in every prefecture, and it is implausible to argue that one cannot be infected with COVID-19 as long as they remain within their own prefecture.

The primary purpose of implementing such measures should be to protect hospitals and patients. Therefore, instead of persisting with travel restrictions that lack scientific backing, even if they were in line with the government's initial policies during the pandemic's onset, it might be more appropriate to revisit and discuss effective infection control measures that do not infringe upon individual privacy and rights, such as distributing free testing kits to students and implementing a self-testing system before internships, as was done during the pandemic in the United Kingdom and other European countries.^8^

At the same time, it is believed that this issue cannot be resolved solely by individual medical schools making changes. As long as such measures continue to be implemented at other universities, it is easy to imagine that deviating from the norm could lead to criticism of not being thorough in COVID-19 countermeasures, thus, the infection control measures must be implemented based on a consensus. Although it has been noted that Japanese organizations are often reluctant to change their responses,^9^ the current management of movement creates psychological barriers to travel, potentially leading to a loss of opportunities for medical students to participate in internships or visit hospitals. Therefore, based on the insights gathered since the onset of the pandemic, Japan's medical schools must develop a flexible approach that allows for internal and external evaluation and adjustment of infection control measures, guarantees learning opportunities for students, and envisages the establishment of testing systems to prevent patient infection, all while adapting to the changes brought by the times.

### Conflicts of Interest

The authors declare that they have no conflict of interest.

## References

[1] Besançon L, Flahault A, Meyerowitz-Katz G (2022). Mobility during the pandemic: how did our movements shape the course of COVID-19?. J Travel Med.

[2] Ehime University School of Medicine. When medical students travel out of prefecture [in Japanese]. 2022 [Cited 2023 April 2]; Available from: bit.ly/3MegPpU.

[3] Japanese Law Translation. The constitution of Japan (Constitution 1946). 1946 [Cited 2023 April 3]; Available from: https://www.japaneselawtranslation.go.jp/ja/laws/view/174/je#je_ch3at13.

[4] Kaneda Y (2023). Regional quota replaced by COVID-19 quota? the dilemma of Japanese medical school admissions.. QJM.

[5] Parmet WE, Goodman RA, Farber A (2005). Individual rights versus the public's health--100 years after Jacobson v. Massachusetts.. N Engl J Med.

[6] Ogasawara F, Yoshida S, Yamane M, Takamatsu K, Arakawa Y, Nishida Y, Komatsu M, Yokoyama A, Yamagishi Y, Kojima K (2023). COVID-19 Cluster in the hematology/respirology ward of a university hospital during the seventh wave of the SARS-CoV-2 pandemic in Japan: a descriptive study.. Intern Med.

[7] Greenhalgh T, Jimenez JL, Prather KA, Tufekci Z, Fisman D, Schooley R (2021). Ten scientific reasons in support of airborne transmission of SARS-CoV-2.. Lancet.

[8] CBS NEWS. At-home COVID tests are a free, easy part of everyday life in the U.K., and the U.S. has taken note. 2021 [Cited 2023 June 5]; Available from: https://www.cbsnews.com/news/at-home-covid-tests-are-a-free-easy-part-of-everyday-life-in-the-uk-and-the-us-has-taken-note/.

[9] Kaneda Y, Ozaki A, Tanimoto T (2023). Rethinking Japan's infallibility principle for a better pandemic response.. Cureus.

